# The Gallery Walk as a method for patient and public involvement in health research: a case study in dementia care

**DOI:** 10.1186/s40900-026-00862-z

**Published:** 2026-03-07

**Authors:** Kübra Annac, Yüce Yilmaz-Aslan, Patrick Brzoska

**Affiliations:** Health Services Research Unit, Faculty of Health/School of Medicine, Witten/Herdecke University, Alfred-Herrhausen-Straße 50, 58455 Witten, Germany

**Keywords:** Gallery Walk, PPI, CBPR, Transdisciplinarity, Dementia

## Abstract

**Background:**

Participatory and transdisciplinary approaches in health research continue to face challenges such as disciplinary silos, power asymmetries, and a lack of accessible formats that enable meaningful dialogue among researchers, practitioners, and individuals with lived experience. This study examines the Gallery Walk as a participatory method in health research, using a workshop on diversity and social position in dementia care to reflect on its potential, limitations, and suitability.

**Methods:**

Twenty participants — researchers, practitioners, individuals living with dementia, and family caregivers — took part in a Gallery Walk across four themed stations. Contributions were documented on flipcharts to support iterative and cumulative discussion, followed by a plenary synthesis.

**Results:**

The Gallery Walk facilitated exchange among heterogeneous groups, supported iterative knowledge building, and generated practice-oriented recommendations. Implementation challenges included the need for careful preparation, skilled facilitation, and adequate resources for documentation and analysis.

**Discussion:**

The use of the Gallery Walk Walk in this workshop demonstrates its significant potential as a participatory and interdisciplinary method in health research. By rotating through thematic stations, engaging with visual content, and iteratively addressing key questions, participants were able to incorporate diverse disciplinary and experiential perspectives. This approach fostered the development of practical recommendations and ensured the meaningful involvement of individuals with dementia and their families. The findings highlight that the Gallery Walk's accessibility and focus on visual elements promote discussion, encourage continuous reflection, and increase transparency by visibly tracking contributions.

**Conclusion:**

The Gallery Walk represents a promising and adaptable format for participatory, transdisciplinary health research when supported by intentional design, inclusive facilitation, and rigorous reporting.

## Abstract (plain language summary)

People with dementia, their families, and health professionals all offer invaluable insights into improving care. However, it can be challenging for these groups to collaborate on equal terms in research. This study explored a creative workshop approach known as the “Gallery Walk”. In this method, participants move around different stations in a room, where ideas and questions are displayed for discussion and written contributions. In our workshop, twenty people—including researchers, practitioners, individuals living with dementia, and family caregivers—exchanged about diversity and social position in dementia care. The Gallery Walk made it easier for everyone to share experiences, learn from each other, and develop practical ideas together. However, some challenges were noted, such as the need for thorough preparation, experienced facilitators, and sufficient time to properly document and analyse the discussions. Overall, the Gallery Walk proved to be a helpful and flexible way to involve people meaningfully in health research, especially when it is thoughtfully planned and led with inclusivity, respect, and clear communication.

## Background

Complex public health challenges—such as chronic disease prevention, mental health promotion, and pandemic preparedness—are inherently multidimensional. They extend well beyond the medical system, encompassing social, cultural, and political dimensions that demand dynamic, cross-sectoral, and integrative solutions [1]. Developing sustainable strategies therefore requires the integration of diverse forms of knowledge and the establishment of interdisciplinary and intersectoral collaborations [2]. Increasingly, it is recognized that such processes cannot be confined to academic contexts alone but must rely on participatory research approaches that connect scientific expertise with the experiential knowledge of affected communities and practitioners [3].

The participation of individuals with lived experience of illness and their social networks has gained increasing attention in recent years. These individuals are not merely participants of research but essential contributors of context-specific and practice-relevant insights [4]. Patient and Public Involvement (PPI) facilitates a deeper understanding of real-world care situations that are often underrepresented in academic research. However, implementing participatory approaches entails multiple challenges—ranging from ensuring equitable access and negotiating diverse disciplinary cultures to addressing power asymmetries between researchers and community participants [5]. While researchers possess institutional and methodological resources, participants typically rely primarily on personal experience. Questions of representation—who is included, whose voices are heard, and who remains excluded—continue to be central to the participatory research agenda [6]. These tensions echo broader critiques within interprofessional and participatory health research, which highlight disparities in disciplinary language, unequal access to power and resources, and the persistent risk of tokenism [7, 8].

Participatory paradigms such as PPI and Community-Based Participatory Research (CBPR) emphasize equitable partnerships between researchers and community members throughout the research process. CBPR, in particular, is characterized by shared decision-making, co-learning, and a strong orientation toward action and social change, with community members actively involved in defining research priorities, interpreting findings, and translating knowledge into practice [9, 10]. These principles align with international policy frameworks—most notably the WHO Global Action Plan on the Public Health Response to Dementia 2017–2025 [11]—which emphasize the inclusion of individuals living with dementia and their caregivers, the reduction of health inequities, and the development of practice-relevant innovations. Translating these values into practice requires research processes that are accessible, structured, and creative – formats that facilitate exchange among diverse stakeholder groups, make individual contributions visible, and lower barriers to participation related to differences in education, language, and cognitive ability [9, 10].

Established participatory methods such as focus groups, World Cafés, and Future Workshops provide valuable opportunities to engage diverse stakeholders and foster dialogue [12]. However, these approaches also have certain limitations: focus groups may privilege dominant voices and elicit socially desirable responses; World Cafés require intensive facilitation and often prioritize breadth over depth; and Future Workshops tend to be resource-intensive and difficult to scale [13, 14].

In dementia research, these methodological limitations are often intensified by condition-specific challenges. Individuals living with dementia may experience reduced attention spans, fatigue, difficulties with verbal expression, and challenges in following fast-paced or abstract discussions. In mixed groups, power asymmetries may be exacerbated when academic or professional participants dominate verbal exchanges, increasing the risk that people with dementia are talked over or withdraw from discussion. These challenges underscore the need for participatory formats that reduce reliance on sustained verbal performance, allow for flexible modes of engagement, and make contributions visible in ways that do not depend solely on spoken language.

Against this backdrop, the Gallery Walk represents a promising complementary format. Combining structure with openness, it fosters equitable participation, creative collaboration, and the systematic integration of perspectives across disciplinary boundaries [15–17].

Originally developed in educational and organizational settings, the method employs a rotating station design and visual documentation that encourage dialogue and iterative sense-making among participants. As individuals move between thematic stations, they collectively build upon previous contributions, facilitating cumulative knowledge generation [18, 19]. Although the Gallery Walk has been successfully applied in schools, companies, and project teams to co-create knowledge and ideas [18, 20], existing studies have predominantly focused on educational contexts with students [16, 17], leaving its potential within participatory health research largely unexplored.

While the Gallery Walk shares certain characteristics with conversational formats such as the World Café—most notably rotation between thematic stations—it differs fundamentally in its epistemic logic and interactional focus. World Café formats primarily rely on facilitated dialogue, with visual documentation serving as a supportive, often secondary element of conversation. In contrast, the Gallery Walk positions visual elements (e.g., cards, flipcharts, written contributions) as the central medium of sense-making. Contributions remain persistently visible across rotations, enabling participants to engage asynchronously by reading, annotating, clustering, or extending prior inputs without requiring continuous verbal exchange. Stations thus function as cumulative visual workspaces rather than conversational tables, allowing knowledge to be co-produced through artifact-based interaction rather than dialogue alone [18, 21].

To the best of our knowledge, this study presents the first systematic application of a Gallery Walk within a transdisciplinary research process in dementia care. By documenting its design, implementation, and participatory outcomes, we aim to illustrate how this method can enhance inclusion, support equitable contribution across stakeholder groups, and generate actionable recommendations for practice and policy in dementia care. Specifically, the study addresses the following research question: *What opportunities and challenges arise when using a Gallery Walk for patient and public involvement in health research*,* and how can this method be effectively implemented in practice?*

In doing so, this article seeks to advance methodological reflection within participatory health research and to encourage further experimentation with the Gallery Walk as an inclusive and generative format for co-producing knowledge in complex health contexts.

## Methods

### Design and setting

A participatory workshop titled “Diversity and Social Position – User Orientation, Participation, and Inclusion in Dementia Care” was conducted on 6 March 2024 in Berlin, Germany as part of the annual Poverty and Health Congress *(Kongress Armut und Gesundheit).* The workshop aimed to explore the still insufficient consideration of diversity and social position in dementia care from multiple perspectives and to develop practice-oriented strategies to strengthen participation.

The event began with two brief expert inputs that provided a shared conceptual framing for subsequent discussions. The first addressed the relationship between dementia and social position [22], while the second introduced the Diversity-On project, funded by the German Federal Joint Committee (G-BA), which focuses on diversity-sensitive approaches in caregiver support [23]. The two expert inputs were delivered by researchers with expertise in dementia care, social inequalities in health, and participatory research. The inputs were deliberately brief and designed to support accessibility rather than to provide comprehensive academic overviews. Content was presented in plain language, with a focus on concrete examples and minimal use of technical terminology. Visual elements were used to support comprehension, and the inputs were framed as an invitation to shared reflection rather than as authoritative lectures.

### Participants

A total of 20 participants took part in the workshop, representing a broad range of stakeholders. Participants were asked to self-identify their affiliation(s); thus, the exact distribution of participants across stakeholder groups cannot be reported. Stakeholder categories represented included researchers, practitioners from dementia careservices, representatives of political bodies, public agencies, and health insurance funds, as well as students, individuals living with dementia, and family caregivers. Recruitment was conducted through congress registration and project networks to ensure diversity in both professional background and lived experience. Individuals living with dementia were attending the congress in a community setting. No clinical inclusion or exclusion criteria (e.g., cognitive screening scores, dementia subtype, or behavioural symptoms) were applied, as the workshop was not designed as a clinical study but as an open participatory format within a public conference.

### Procedure: The Gallery Walk

To facilitate structured yet flexible dialogue, a Gallery Walk format was employed. Four thematic stations addressed key dimensions of participation in dementia care:

1. **Social position**: How can attention to the social position of individuals living with dementia and their caregivers be strengthened?
2. **User orientation**: How can user orientation in dementia services be improved?
3. **Participation and co-creation**: How can individuals living with dementia and their caregivers be reached and involved in participatory projects?
4. **Continuity of participation**: How can the ongoing involvement of individuals living with dementia and their caregivers in research and care initiatives be sustained?

Each station was equipped with a prepared flipchart and guided by a leading question. To deepen reflection and ensure comparability, all stations shared three discussion prompts:

1. What needs to be done (or is already being done)?
2. How can it be implemented (or how is it currently implemented)?
3. Who should take responsibility (or who already does)?

Participants were randomly assigned to small groups, ensuring that each station included a combination of professional and lived-experience perspectives, each beginning at a different station. Facilitators – i.e., individuals responsible for moderating the group discussions — supported the discussions and documented key points on moderation cards, which were visibly attached to the flipcharts. The facilitators were members of the research team with prior experience in participatory research and dementia-sensitive communication. Before the workshop, facilitators received a structured briefing from KA, the lead project staff member, who was also responsible for the overall design and organization of the workshop. The briefing covered the aims of the Gallery Walk, principles of inclusive moderation, strategies for managing mixed groups, and techniques to support participation by individuals with cognitive or linguistic challenges. Facilitators supported understanding by paraphrasing contributions, avoiding technical terminology, and relating discussions to everyday care experiences.

**Fig. 1:**
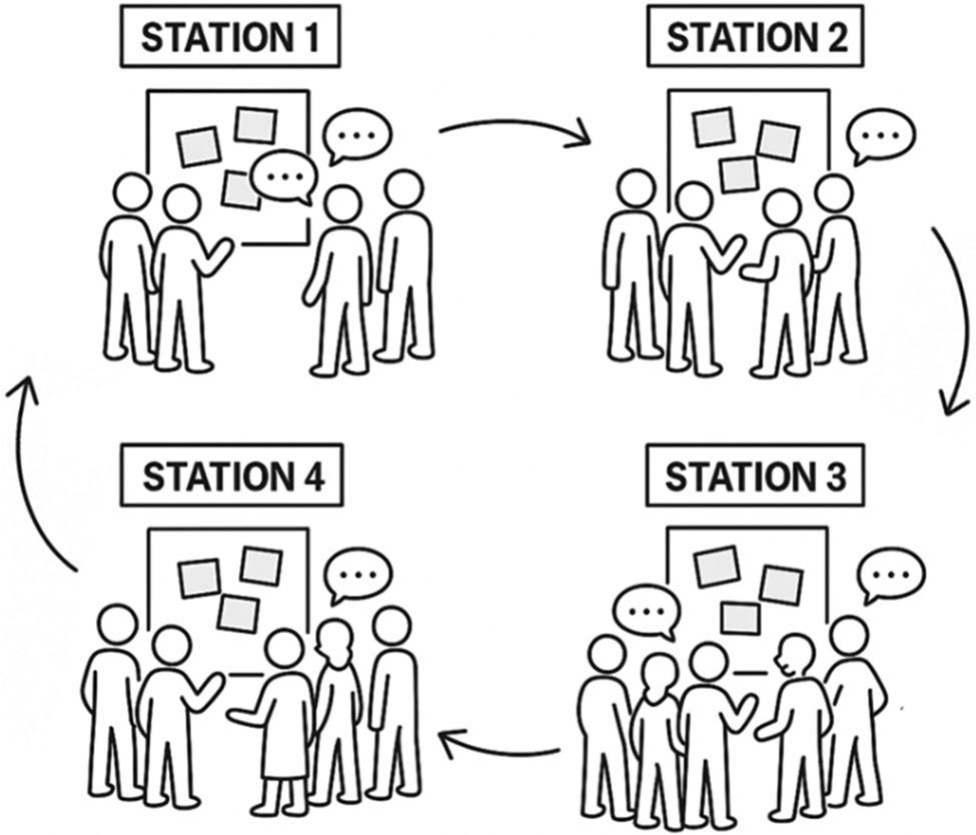
Schematic representation of a Gallery Walk with four stations

Unlike World Café tables, which are typically structured around ongoing facilitated conversations, each Gallery Walk station was designed as a visually anchored working surface. The primary mode of interaction was engagement with existing written and visual contributions, which participants could extend, rearrange, or supplement verbally or in writing. This design allowed meaningful participation even when individuals chose not to engage continuously in group conversation. After approximately ten minutes, groups rotated to the next station, allowing them to build cumulatively on prior contributions (Fig. 1). This iterative and visual process encouraged the integration of diverse perspectives and helped mitigate dominance effects commonly observed in traditional group discussions. After approximately forty minutes, all groups had visited each station. The workshop concluded with a plenary synthesis session in which the accumulated ideas were reviewed, collectively reflected upon, and consolidated into practice-oriented recommendations.

### Data collection and analysis

Data relevant to the research question on opportunities, challenges, and implementation of the Gallery Walk were derived from the visual materials produced during the workshop (flipcharts, moderation cards) and from facilitators’ reflective field notes on participation dynamics and process quality. All materials generated during the Gallery Walk were digitized immediately after the workshop. A rapid content analysis approach [24] was employed to identify recurring themes, gaps, and actionable strategies for strengthening participation among individuals living with dementia and their relatives. The analytic process comprised three steps: (1) inductive coding of visual material, (2) thematic clustering, and (3) synthesis of cross-cutting strategies. Facilitator reflections and field notes were integrated to support interpretation, with particular attention to both process quality (e.g., inclusivity, interaction dynamics) and outcome relevance (e.g., feasibility of proposed actions). The systematic post-workshop analysis of the visual materials was conducted by members of the research team with experience in qualitative and participatory methods. However, analytic reflection was not limited to this post hoc phase. During the concluding plenary synthesis, participants—including individuals with lived experience and people living with dementia—were invited to jointly review and reflect on the accumulated visual material, discuss emerging themes, and identify preliminary priorities and points of convergence across stations.

### Ethical and inclusivity considerations

The workshop was conducted in accordance with the principles of participatory ethics, emphasizing inclusivity, transparency, and psychological safety. The materials and station prompts were developed by the research team for use in an open congress workshop aimed at bringing together a broad range of stakeholder groups and conceptualized as a shared participatory format to facilitate collaboration and exchange across diverse professional and experiential perspectives. All written and visual materials were prepared in plain language and supplemented with pictorial cues to enhance accessibility across different educational levels, linguistic backgrounds, and cognitive capacities.

The GRIPP2 framework [25] guided the reporting of the aims, methods, and outcomes of PPI in our research. In line with the NIHR INCLUDE framework [26], specific efforts were made to identify and reduce barriers for under-served groups, including individuals with limited health literacy, non-native German speakers, and people with mild to moderate cognitive impairment. The participation of individuals living with dementia was facilitated through assisted moderation, shorter rotation intervals, and the option to contribute either verbally or visually. Key terms were explained verbally and visually, and facilitators used slow pacing, repetition, and paraphrasing where needed. Visual materials were displayed at eye level and remained visible throughout the workshop to support orientation and recall.

## Results

The application of the Gallery Walk in the workshop “Diversity and Social Position – User Orientation, Participation, and Inclusion in Dementia Care” revealed several key mechanisms and methodological potentials that fostered engagement, facilitated knowledge integration, and promoted participatory exchange.

### Mechanisms supporting engagement and integration

The initial expert inputs were essential for establishing a shared conceptual framing. By providing a common knowledge base and clarifying the significance of diversity and social position in dementia care, these introductory presentations prepared participants for focused and informed engagement during the Gallery Walk.

The heterogeneous composition of the small groups—comprising researchers, practitioners, policymakers, students, individuals living with dementia, and family caregivers—enabled rich, interdisciplinary dialogue. Diverse professional and experiential perspectives were directly integrated into the discussions, generating both theoretically grounded and practice-oriented insights across the four thematic stations. The rotating format and the presence of visible, pre-existing contributions supported focused engagement by allowing participants to respond to and build on documented ideas. This facilitated meaningful interaction without requiring extended or continuous verbal discussion.

The rotating structure of the Gallery Walk emerged as a particularly effective design feature. As groups progressed through the stations, participants built iteratively on the contributions of previous groups. This cumulative process deepened thematic exploration, facilitated the integration of perspectives across topics, and fostered creative connections between structural, ethical, and practical dimensions of participation. The visual documentation on cards and flipcharts enhanced transparency, allowing participants to trace, reflect upon, and expand ongoing discussions in real time. Importantly, the cumulative development of content did not depend on conversational continuity within fixed groups, as would be typical in World Café formats. Instead, knowledge integration emerged through successive engagement with the same visual elements across rotating groups, allowing perspectives to accumulate independently of who was present at a given moment.

The multimodal engagement—combining written, visual, and physical interaction—helped to reduce participation barriers for individuals living with dementia and for participants with different levels of professional or linguistic familiarity. The visual and physical environment played a central role in supporting integration and engagement. Large-format flipcharts, clearly structured prompts, and color-coded moderation cards allowed participants to orient themselves quickly and engage with content through reading, pointing, or adding brief written notes. Physical movement between stations helped maintain attention and reduced cognitive fatigue. While verbal interaction remained part of the process, the availability of visible reference points reduced reliance on memory and spontaneous speech, thereby supporting participants who experienced difficulties with verbal expression. The visible and interactive character of the method promoted equal opportunities for expression, supporting inclusion and active co-creation.

The role of facilitation was critical in this regard: effective moderation at each station ensured that diverse voices were acknowledged and that discussions remained dynamic and respectful. Facilitators actively monitored interaction dynamics to ensure that individuals living with dementia were not talked over. This included explicitly inviting their perspectives, allowing additional time for responses, and redirecting discussions when dominant voices emerged. During the plenary synthesis, facilitators referenced written contributions rather than individual speakers, ensuring that ideas generated by people with dementia remained visible and influential regardless of who articulated them verbally.

The concluding plenary synthesis added an important reflective dimension. The concluding plenary synthesis provided an opportunity to jointly review and reflect on the accumulated visual material and to identify preliminary priorities and points of convergence across stations. This final step transformed the participatory exchange into a set of concrete, practice-oriented proposals for enhancing participation and equity in dementia care.

### Implementation challenges

Despite its clear benefits, several challenges emerged during the implementation of the Gallery Walk. Achieving a balance between inclusivity and analytical depth required careful preparation, clear instructions, and skilled moderation to ensure that all participants could contribute meaningfully while maintaining conceptual coherence. The limited duration of the workshop constrained opportunities for in-depth reflection on complex socio-structural issues and the interconnections between themes, which would have benefited from extended discussion or follow-up sessions. In addition, the systematic capture, digitization, and analysis of the visual materials generated during the Gallery Walk demanded substantial time and coordination. This highlighted the need for sufficient resource allocation and methodological support when applying the Gallery Walk in participatory research contexts.

## Discussion

### Methodological potentials of the Gallery Walk

The use of the Gallery Walk in this workshop illustrates the substantial methodological potential of this approach for participatory and interdisciplinary health research. By rotating between thematic stations, engaging with visual documentation on flipcharts, and iteratively exploring key questions, participants were able to integrate a wide range of disciplinary and experiential perspectives. This process supported the development of practice-oriented recommendations and facilitated the equitable involvement of individuals living with dementia and their relatives. The findings suggest that the Gallery Walk – through its low-threshold accessibility and emphasis on visual content – stimulates discussion, encourages ongoing reflection, and enhances transparency by making the accumulation of contributions visible [21].

The method proved particularly effective in engaging participants with diverse educational backgrounds and varying levels of prior experience. The combination of written, visual, and tactile interaction—using cards, markers, and pinboards—encouraged active, low-barrier participation. The visible documentation of ideas and the physical movement between stations generated an embodied learning and reflection process that appealed even to those who might contribute less actively in purely verbal formats such as focus groups or World Cafés [13, 14]. In contrast to these conversation-centered approaches, the Gallery Walk accommodates multiple modes of expression beyond spoken language, thereby facilitating participation among individuals with cognitive impairments or limited linguistic or academic experience. The Gallery Walk does not depend primarily on verbal interaction. Participation is enabled through written and visual engagement, including reading and arranging cards, adding brief written contributions, and using colors or symbols on the flipcharts. Verbal input is optional rather than required, as persistent visual elements allow participants to engage with and contribute to the discussion without continuous spoken interaction. This design can reduce pressure on verbal expression and support participation at an individual pace, provided that materials and facilitation are dementia-sensitive [18].

Compared to other participatory formats, such as focus groups or World Cafés, the Gallery Walk offers distinct methodological advantages. It supports distributed participation rather than turn-taking, mitigates dominance effects, and externalizes group thinking through artifact-based sense-making [15]. Dominance effects were mitigated through a combination of structural and facilitative mechanisms. Time-limited rotations prevented prolonged monopolization of discussion, while the centrality of written and visual elements shifted attention away from individual speakers toward collectively produced content. Facilitation further contributed by redirecting focus to existing contributions and inviting input from quieter participants, thereby reducing the influence of hierarchical or professional status on group dynamics. Its visual and physical components allow participants to collectively “see” the development of ideas and the relationships between themes, thereby enhancing conceptual coherence and cumulative knowledge building. Moreover, the structured yet flexible rotation design enables multiple topics to be explored within a limited timeframe, generating breadth without compromising the potential for depth. Taken together, these features position the Gallery Walk as a valuable complement to existing participatory techniques in health research, particularly when inclusivity, creativity, and rapid synthesis are prioritized [27].

The findings align closely with the principles of CBPR, which emphasize co-learning, capacity building, and shared ownership [9]. In condensed workshop settings, the Gallery Walk operationalizes these principles by distributing voice, making knowledge visible, and enabling iterative synthesis. In doing so, the method supports a central aim of transdisciplinary health research — the integration of scientific, professional, and experiential forms of knowledge [2]. It also resonates with human-centred design (HCD) approaches widely applied in global health contexts [28, 29], which emphasize iterative ideation, tangible co-creation, and the development of context-sensitive solutions.

### Ethics, equity, and reporting

Inclusive research requires sustained attention to issues of representation, burden, and benefit. The workshop findings underscore the importance of ethical reflexivity and transparency in participatory processes. The GRIPP2 framework [25] provides a valuable structure for reporting the aims, processes, and impacts of participant involvement, thereby strengthening reproducibility and accountability. Likewise, the NIHR INCLUDE roadmap [26] offers practical guidance for identifying and mitigating exclusion risks — particularly relevant in dementia research, where cognitive, linguistic, and socio-economic barriers intersect. The multimodal and flexible nature of the Gallery Walk proved useful in lowering such barriers and promoting equitable engagement, while also highlighting the ongoing need for methodological vigilance to ensure that diverse voices are represented meaningfully rather than symbolically.

### Practical and policy relevance

The workshop’s findings also have clear implications for policy and practice. They align with the WHO Global Action Plan [11], which underscores person-centred care, caregiver support, and the meaningful inclusion of individuals living with dementia in research and policy-making. The Gallery Walk offers a pragmatic and scalable mechanism for translating these global objectives into locally grounded, co-produced strategies. By combining participatory ethics with structured facilitation, the method helps identify context-specific needs and actionable entry points for innovation in dementia care and related fields.

Given the resource demands associated with data collection and analysis in face-to-face Gallery Walks, digital and hybrid adaptations represent a promising methodological development. Online tools such as Miro^1^ or Mural^2^ enable remote participation, real-time visualization, and streamlined documentation, potentially extending inclusivity to participants who cannot attend in person. Evidence from digital education and participatory design research [30, 31] suggests that virtual Gallery Walks can sustain engagement and creative collaboration when accessibility and facilitation are carefully designed. Integrating digital components may therefore enhance both the scalability and sustainability of future participatory health research initiatives. At the same time, however, digital or hybrid Gallery Walk formats may also introduce new barriers, particularly for individuals with limited digital literacy, restricted access to technology, or cognitive impairments [30, 31]. Digital adaptations should therefore be considered cautiously and accompanied by appropriate support measures to avoid reproducing forms of exclusion.

### Limitations

This study draws on a single workshop conducted in a conference setting with a relatively small, self-selecting group of participants. Stakeholder affiliation was recorded only as category membership (self-identification) and not quantified; thus, we cannot report the exact distribution of participants across stakeholder groups. In addition, participation took place in a conference context, which may have led to self-selection of individuals who were already relatively empowered or experienced in participatory settings. This may limit the transferability of findings to less experienced or more marginalized groups. Participants were invited to reflect on and provide feedback regarding their experience with the Gallery Walk during the concluding plenary discussion. However, no systematic or structured post-workshop feedback was collected. Future applications of the method may benefit from incorporating dedicated feedback instruments to more comprehensively capture participant experiences and suggestions. Although the findings demonstrate clear methodological and participatory benefits, they should be regarded as exploratory rather than generalizable. Future research should systematically compare the Gallery Walk with alternative participatory methods in terms of participant experience, perceived inclusion, satisfaction, and the uptake of generated recommendations. Longitudinal studies could further examine whether the method contributes to sustained changes in policy or practice, as well as how it may be adapted for use in different cultural or institutional contexts.

### Practical implications

Building on the findings of the workshop, the following practical implications summarize key considerations for applying the Gallery Walk as a participatory method in similar contexts. These implications are not presented as empirical results but as practice-oriented guidance derived from the observed opportunities and challenges (Table 1).

**Table 1:** Practical recommendations derived from the Gallery Walk implementation

Recommendation	Context / rationale	Application in this workshop
Develop predefined thematic stations	Clear structure is required to support engagement in heterogeneous groups.	Four predefined thematic stations were developed, each with one guiding question and three standardized prompts to ensure comparability across stations.
Use short, accessible guiding questions	Accessible language lowers barriers for participation across stakeholder groups.	Concepts such as “participation” and “social position” were translated into experience-based questions discussed at each station.
Ensure skilled and inclusive facilitation	Facilitation is central to balancing participation and supporting heterogeneous groups.	Facilitators supported discussions, invited quieter participants to contribute, paraphrased inputs in plain language, and documented contributions on cards.
Prioritize visual documentation over verbal exchange	Persistent visual elements enable participation beyond spoken interaction.	All contributions were written on moderation cards and flipcharts and remained visible throughout the workshop, enabling cumulative engagement across rotations.
Structure rotations to enable cumulative knowledge building	Iterative engagement supports integration of perspectives across groups.	Groups rotated between stations and built on existing visual material rather than restarting discussions.
Provide materials in plain language and multiple formats	The use of minimal and/or plain language supports participation of individuals with cognitive or linguistic barriers.	Plain language was used across all written materials, with short sentences and clearly structured prompts, complemented by visual structuring (e.g., spacing, colors, headings) and optional use of symbols to support orientation and comprehension.
Use visualization and physical interaction	Visualization and physical interaction encourage creativity and engagement across educational backgrounds.	Flipcharts and moderation cards were used at each station, allowing participants to read, add, and rearrange contributions. All materials were displayed visibly within the room, and movement between stations supported physical engagement with the content.
Integrate a concluding plenary reflection	Collective reflection supports synthesis and validation of outcomes.	The workshop ended with a plenary session in which participants jointly reviewed the visual material, reflected on key themes, and identified preliminary priorities.
Allocate sufficient resources for documentation and analysis	Visual outputs require post-workshop processing.	All flipcharts and cards were digitized after the workshop and formed the basis for subsequent thematic analysis.

## Conclusions

Under conditions of rigorous methodological design and skilled facilitation, the Gallery Walk constitutes a low-threshold and highly engaging approach to participatory and transdisciplinary health research. The workshop demonstrated that the method integrates diverse disciplinary and experiential perspectives, promotes creative problem-solving, and supports the equitable participation of individuals with lived experience, including those facing cognitive or linguistic barriers. By combining rotation between thematic stations with visual documentation, the Gallery Walk stimulates iterative dialogue, fosters cumulative understanding, and translates complex discussions into practical, actionable strategies.

The strengths of the method lie in its flexibility and inclusivity. It can be adapted to different group sizes, topics, and settings within health research and practice, ranging from small-scale co-design sessions to large participatory conferences. Its emphasis on visualization and physical interaction provides an accessible entry point for participants with varied educational and professional backgrounds, making it particularly suitable for contexts — such as dementia care — where conventional verbal formats may constrain engagement. At the same time, successful implementation requires methodological rigor and careful preparation. Integrating ethical and inclusivity frameworks — such as GRIPP2 for transparent reporting of PPI [25] and NIHR INCLUDE for inclusive recruitment and participation planning [26] — can further strengthen scientific rigor and equity. These frameworks help ensure that participation is not merely symbolic but contributes meaningfully to the generation of relevant, implementable outcomes.

Linking the method to broader frameworks — such as the WHO Global Action Plan on the Public Health Response to Dementia (2017–2025) [11] — positions it as a scalable and pragmatic tool for localizing international policy objectives related to person-centred care, caregiver involvement, and inclusive innovation. Beyond dementia care, the Gallery Walk holds potential for a wide range of participatory health research contexts in which shared understanding, creativity, and equitable collaboration are essential.

In summary, the Gallery Walk represents a promising, flexible, and participatory method that effectively bridges research, practice, and policy. Its structured yet open design – combining visual documentation with multimodal engagement – fosters interdisciplinary dialogue, creative problem-solving, and the equitable inclusion of individuals with lived experience. Particularly suited to transdisciplinary projects addressing complex health challenges, the method enables genuine co-production and the development of actionable, practice-relevant insights. Nonetheless, its successful implementation depends on methodological rigor, adequate resources, and skilled facilitation. When thoughtfully designed and transparently reported, the Gallery Walk can make a meaningful contribution to advancing inclusive, socially responsive, and impact-oriented health research.

## Data Availability

All data generated or analysed during this methodology study are included in this published article. The datasets generated and analysed during the workshop are available in Annac K, Franz D, Basyigit M, Öztürk S, Tezcan-Güntekin H, Örs E, Hofrichter P, Kreinhöfer D, Rutenkröger A, Kuhn C, Yilmaz Aslan Y, Brzoska P. Diversität und soziale Lage – Workshop zur Nutzerorientierung, Partizipation und Teilhabe im Demenzbereich. 29. Kongress Armut und Gesundheit, 05.-06.03.2024. 2024. Available: https://www.armut-und-gesundheit.de/fileadmin/user_upload/Kongress/Kongress_2024/Dokumentation_2024/334.pdf.

## Footnotes

1
https://miro.com/.

2
https://www.mural.co/.
